# Constitutive depletion of *Slc34a2*/NaPi-IIb in rats causes perinatal mortality

**DOI:** 10.1038/s41598-021-86874-z

**Published:** 2021-04-12

**Authors:** Eva Maria Pastor-Arroyo, Josep M. Monné Rodriguez, Giovanni Pellegrini, Carla Bettoni, Moshe Levi, Nati Hernando, Carsten A. Wagner

**Affiliations:** 1 Institute of Physiology, University of Zürich, Winterthurerstrasse 190, 8057 Zurich, Switzerland; 2 Laboratory for Animal Model Pathology (LAMP), Institute of Veterinary Pathology, Vetsuisse Faculty, University of Zurich, Winterthurerstrasse 268, 8057 Zurich, Switzerland; 3 Department of Biochemistry and Molecular and Cellular Biology, Georgetown University, Washington, DC USA

**Keywords:** Physiology, Calcium and vitamin D, Kidney, Metabolism

## Abstract

Absorption of dietary phosphate (Pi) across intestinal epithelia is a regulated process mediated by transcellular and paracellular pathways. Although hyperphosphatemia is a risk factor for the development of cardiovascular disease, the amount of ingested Pi in a typical Western diet is above physiological needs. While blocking intestinal absorption has been suggested as a therapeutic approach to prevent hyperphosphatemia, a complete picture regarding the identity and regulation of the mechanism(s) responsible for intestinal absorption of Pi is missing. The Na^+^/Pi cotransporter NaPi-IIb is a secondary active transporter encoded by the *Slc34a2* gene. This transporter has a wide tissue distribution and within the intestinal tract is located at the apical membrane of epithelial cells. Based on mouse models deficient in NaPi-IIb, this cotransporter is assumed to mediate the bulk of active intestinal absorption of Pi. However, whether or not this is also applicable to humans is unknown, since human patients with inactivating mutations in *SLC34A2* have not been reported to suffer from Pi depletion. Thus, mice may not be the most appropriate experimental model for the translation of intestinal Pi handling to humans. Here, we describe the generation of a rat model with Crispr/Cas-driven constitutive depletion of *Slc34a2*. *Slc34a2* heterozygous rats were indistinguishable from wild type animals under standard dietary conditions as well as upon 3 days feeding on low Pi. However, unlike in humans, homozygosity resulted in perinatal lethality.

## Introduction

Dietary inorganic phosphate (Pi) is mainly absorbed along the small intestine, and depending on the type of diet absorption represents 40–60% of total ingested phosphate (for review see^1^). Absorption of Pi along the small intestine proceeds via two independent pathways: an active component, saturable at low luminal concentrations of Pi (Km in the µM range), and a poorly understood passive component that increases linearly with increasing concentrations of luminal Pi^2–8^ (for review see^1^). Active transport is mediated to a major extent by NaPi-IIb/*Slc34a2*, a transporter that uses the Na^+^ gradient as a driving force for the uptake of Pi, though other Na^+^-dependent Pi transporters such as PiT-1 and PiT-2, as well as Na^+^-independent mechanism(s), including a H^+^-driven transporter, have also been proposed to participate in the process^6,9–12^. Absorbed Pi is then distributed in skeletal and soft tissues, according to the needs of the organism, whereas excess Pi is excreted by the kidneys, the organs classically considered to be responsible for the fine control of plasma Pi levels. Only a small percentage of total Pi circulates in plasma.

NaPi-IIb/*Slc34a2*, the second identified member of the *Slc34* family of Na^+^/Pi cotransporters, is a transmembrane protein of about 700 amino acids predicted to fully span 8 times the apical membrane of epithelial cells^13,14^. NaPi-IIb has a wide tissue distribution: in addition to enterocytes, its mRNA expression has been reported in salivary glands, uterus, mammary glands, testis, thyroid gland, kidney, heart and placenta^15^. Despite this wide pattern of expression, mutations of *SLC34A2* in humans are only causing pulmonary alveolar microlithiasis (PAM: OMIM 265100)^16,17^, with some data also suggesting a role in the development of testicular microlithisasis^16^ and tricuspid valve calcification^18^. In addition, *SLC34A2* has been implicated in the progression of several malignant tumors based on the observation that its mRNA is overexpressed in carcinomas affecting thyroid, lung, ovary, breast, liver, kidney and intestinal tract (for review see^19^). However, studies describing the presence of the transporter at the protein level are mostly limited to intestine, though protein expression in lung, liver and kidney has been reported^20–22^. Unlike NaPi-IIb, expression of the other two members of the Slc34 family (NaPi-IIa/*Slc34a1* and NaPi-IIc/*Slc34a3*) is rather restricted to kidney^23,24^, and their mutations in humans associate with several hypophosphatemic syndromes, including infantile hypercalcemia type 2 (HCINF2: OMIM 616963)^25,26^ and hereditary hypophosphatemic rickets with hypercalciuria (HHRH: OMIM 609826)^27–29^, respectively.

Expression of intestinal and renal Pi transporters is under endocrine control. Thus, 1,25(OH)2 vitamin D_3_ upregulates the expression of NaPi-IIb in the gut epithelia and therefore stimulates intestinal absorption of Pi^7,30^. In contrast, fibroblast growth factor 23 (FGF23) and parathyroid hormone (PTH) downregulate the renal expression of NaPi-IIa and NaPi-IIc, thus reducing renal reabsorption of Pi and promoting a phosphaturic response (for review see^31^). PTH and FGF23 do not directly affect intestinal Pi absorption but may act via altered 1,25(OH)2 vitamin D_3_ levels.

The expression profile of NaPi-IIb along the intestinal tract is species specific: the transporter is found in the initial segments of the small intestine in rats^32,33^, whereas its maximal expression in mice has been documented in the ileum^33–35^. This pattern overlaps with the intestinal segments exhibiting the highest rate of active transport of Pi, i.e. jejunum in rats^5,36^, and ileum in mice^33–35^. A higher contribution of jejunum than ileum to intestinal Pi absorption has also been suggested in humans^8,37^. A further striking difference across species is the physiological consequence of mutations/ablation of the cotransporters’ genes. Thus, numerous homozygous or compound heterozygous mutations of *SLC34A2* have been reported in human patients affected by PAM, a disease characterized by the intra-alveolar deposition of mineral crystals that is probably the consequence of the failure of the mutated cotransporter to clear Pi from the alveolar lumen^16,17^. Although the effect of these mutations in the sorting and/or activity of the cotransporter has not been analyzed, some of them (including non-sense substitutions or truncations within exons 1–3) are expected to produce severely truncated forms of the protein hardly expected to display any transport activity^16,38^. Despite this fact, PAM has been diagnosed not only in newborns^39^ and infants^40^ but more often in adult and even elderly persons^41^, suggesting that functional inactivation of NaPi-IIb in humans is not lethal. In contrast, constitutive ablation of *Slc34a2* causes embryonic lethality in mice^42^, though its conditional depletion replicates the human PAM phenotype resulting in impaired alveolar absorption of Pi and microlithiasis^17,43,44^. These differences between human and mice raised questions as to whether mice are the proper experimental model from which the role of intestinal NaPi-IIb in Pi balance can be translated to humans. Here, we describe the generation of a rat model with constitutive depletion of *Slc34a2*.

## Results

### Breeding of *Slc34a2* heterozygous rats produced no homozygous offspring

**Figure 1:**
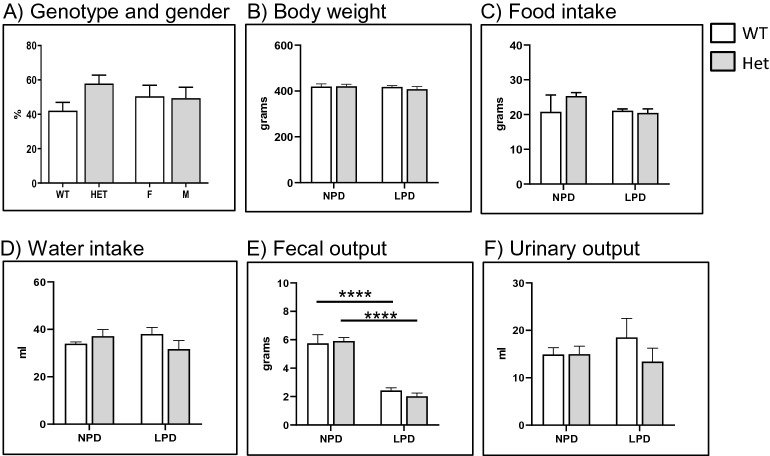
(**A**) Genotype and gender from pups from 9 litters born from two independent heterozygous *Slc34a2* breedings. (**B**) body weight, (**C**) food intake, (**D**) water intake, (**E**) fecal output and (**F**) urinary output of wild type (n = 4) and heterozygous (n = 5) *Slc34a2* rats fed standard chow (NPD) or challenged for 3 days with low Pi diet (LPD). Data are presented as means ± SEM.

Nine litters obtained from two independent heterozygous breedings produced only wild type and heterozygous pups at a ratio of 41% wild types and 59% heterozygous, with males and females born at similar ratios (Fig. 1A). We have previously reported that challenging *Slc34a2* deficient mice with low dietary Pi reveals hormonal and electrolyte alterations that were otherwise not observed in mice fed standard diets^45^. Therefore, we compared several parameters in wild type and *Slc34a2* heterozygous rats fed standard chow as well as upon three days on low dietary Pi. No differences between littermates of both genotypes were found with regard to body weight, food intake, water intake and fecal/urinary outputs (Fig. 1B–F). Except for the fecal output that was reduced in rats fed low Pi, all other parameters were similar in both genotypes and dietary conditions.

### *Slc34a2* heterozygous rats are indistinguishable from wild types with regard to several parameters related to phosphate balance

**Figure 2:**
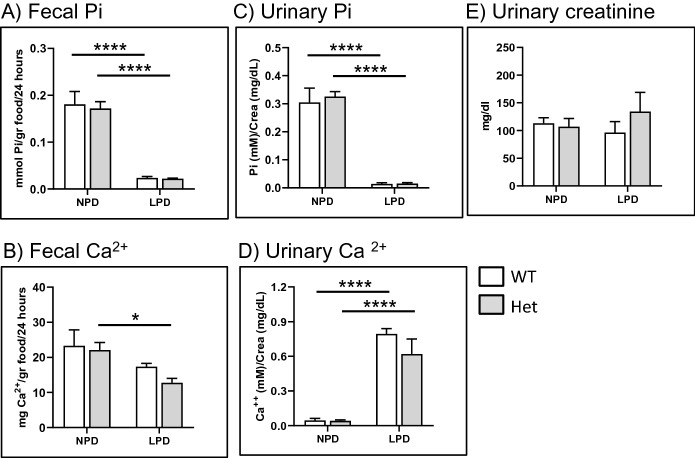
(**A**) Fecal Pi, (**B**) fecal Ca^2+^, (**C**) urinary Pi, (**D**) urinary Ca^2+^ and (**E**) urinary creatinine of wild type (n = 4) and heterozygous (n = 5) *Slc34a2* rats fed standard chow (NPD) or challenged for 3 days with low Pi diet (LPD). Data are presented as means ± SEM. Statistical significance was calculated by ANOVA-Bonferroni. *P ≤ 0.05 and ****P ≤ 0.0001.

Fecal excretion of Pi was comparable in wild type and heterozygous rats fed normal chow, and in both genotypes it reflected the content of Pi in the food, being markedly reduced upon Pi restriction (Fig. 2A). Under normal dietary conditions, the fecal excretion of Ca^2+^ was also comparable in both genotypes, and both groups showed a tendency for reduced excretion upon feeding low Pi, though the difference was significant only in heterozygous (Fig. 2B).

The urinary excretion of Pi (Fig. 2C) and Ca^2+^ (Fig. 2D) were also similar in wild type and heterozygous rats. Furthermore, both parameters adjusted to changes in dietary Pi as expected, i.e. excretion of Pi was reduced whereas excretion of Ca^2+^ was increased upon feeding low Pi, with the magnitude of these changes been comparable in both genotypes. Urinary creatinine values were also comparable in wild type and heterozygous rats, both under normal dietary condition as well as after challenging with low Pi (Fig. 2E). The absence of differences in fecal and urinary excretion of Pi between wild hype and heterozygous rats is in agreement with our findings in mice, where increased fecal excretion and reduced urinary output of Pi were observed only *Slc34a2* homozygous animals^44^.

**Figure 3:**
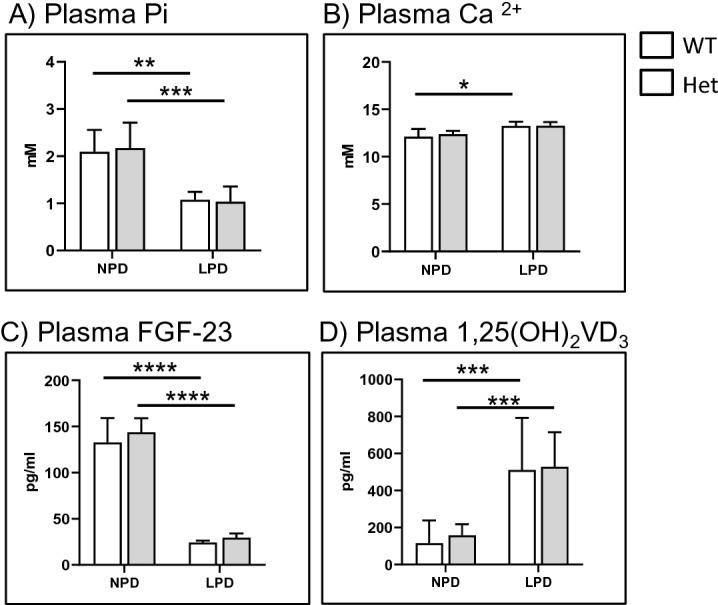
(**A**) Plasma Pi, (**B**) plasma Ca^2+^, (**C**) plasma intact FGF-23 and (**D**) plasma 1,25(OH)_2_ vitamin D_3_ of wild type (n = 4) and heterozygous (n = 5) *Slc34a2* rats fed standard chow (NPD) or challenged for 3 days with low Pi diet (LPD). Data are presented as means ± SEM. Statistical significance was calculated by ANOVA-Bonferroni. *P ≤ 0.05, **P ≤ 0.01, ***P ≤ 0.001, and ****P ≤ 0.0001.

Under both dietary conditions, the plasma levels of Pi (Fig. 3A) and Ca^2+^ (Fig. 3B) were similar in wild type and heterozygous rats. The expected reduction in plasma Pi concentration was observed in all animals after feeding low dietary Pi, whereas dietary Pi restriction resulted in a small but significant increase in plasma Ca^2+^ only in wild type rats. No differences between genotypes regarding plasma levels of intact FGF23 (Fig. 3C) and 1,25(OH)_2_ vitamin D_3_ (Fig. 3D) were detected, similar to our previous findings in mice where FGF23 levels were reduced only in homozygous females while 1,25(OH)_2_ vitamin D_3_ levels were indistinguishable even between wild type and homozygous mice^44^. In both groups, FGF23 was properly and comparably reduced upon feeding low Pi, whereas as expected the plasma levels of 1,25(OH)_2_ vitamin D_3_ were increased upon dietary Pi restriction.

**Figure 4:**
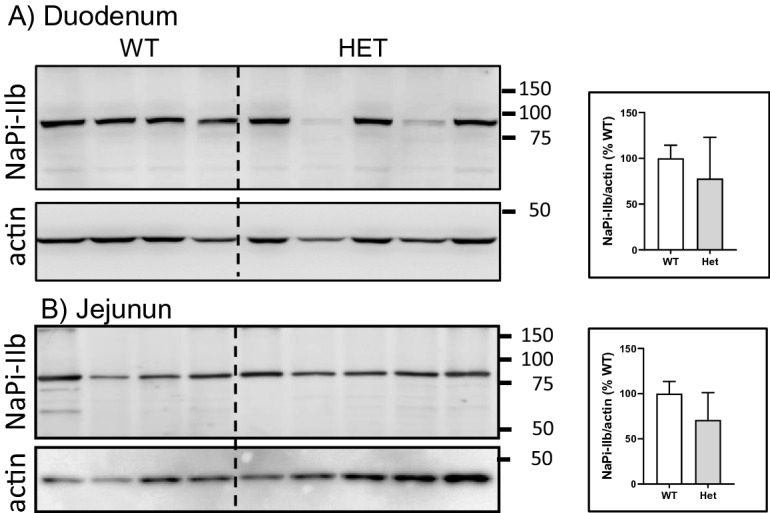
Expression of NaPi-IIb in total membranes isolated from mucosa of (**A**) duodenum and (**B**) jejunum from wild type (n = 4) and heterozygous (n = 5) *Slc34a2* rats challenged for 3 days with low Pi diet. The abundance of the cotransporter was normalize to the expression of actin. The ratio in WT rats was considered as 100%.

The absence of differences in fecal, urinary and hormonal parameters correlated with similar expression of NaPi-IIb in total membranes isolated from duodenum (Fig. 4A) and jejunum (Fig. 4B) from wild type and heterozygous rats challenged for three days with low dietary Pi. Original Western blot images are shown in supplementary Fig. 1.

### *Slc34a2* homozygous embryos are detected at the expected Mendelian ratio at stage E18, but have a reduced body weight

**Figure 5:**
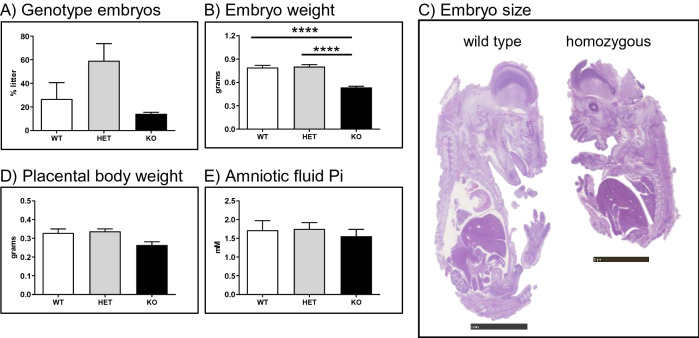
(**A**) Genotype of E18 embryos born from 3 heterozygous breedings. Embryonic (**B**) body weight, (**C**) representative images, (**D**) placental body weight and (**E**) amniotic fluid Pi concentration. Data are presented as means ± SEM. Scale bars in (**C**): 5 mm. Statistical significance was calculated by ANOVA-Bonferroni. ****P ≤ 0.0001.

In order to analyse why we did not detect homozygous mutant rats among the newborn pups, we examined whether homozygous pups could be detected in utero and whether the cause of death could be established. Three heterozygous females crossed with heterozygous males produced wild type, heterozygous and homozygous embryos (stage E18) close to the expected Mendelian ratio (Fig. 5A). However, at this embryonic stage homozygous embryos had significantly smaller body weight than wild type and heterozygous littermates (Fig. 5B) and were easily recognized by eye (Fig. 5C). Placental weight also tended to be smaller in homozygous embryos, but the differences was not significant (Fig. 5D), whereas similar concentrations of Pi were detected in the amniotic fluid of all genotypes (Fig. 5E).

### Organ morphology is similar in wild type and *Slc34a2* homozygous E18 embryos

**Figure 6:**
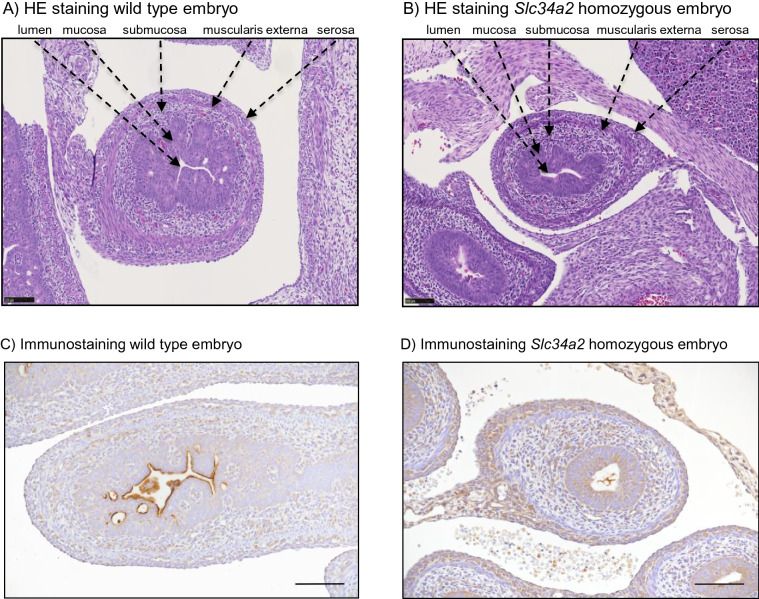
Intestines from (**A**,**C**) wild type and (**B**,**D**) *Slc34a2* homozygous E18 embryos stained with (**A**,**B**) H&E or with (**C**,**D**) a NaPi-IIb antibody. Scale bars: 100 µm in (**A**,**B**); 50 µm in (**C**,**D**).

Although homozygous embryos were markedly smaller compared to the wild type littermates, no macroscopical or histological anomalies were identified in intestines, lungs, liver, pancreas and kidneys. Figure 6 shows intestinal sections of wild type (A) and homozygous *Slc34a2* embryos (B) stained with H&E. In samples from both genotypes it was possible to differentiate cell layers corresponding to muscularis externa, submucosa and mucosa. Furthermore, villi morphogenesis was evident in intestines of wild type and homozygous embryos. Immunohistochemical examination for NaPi-IIb showed a strong signal in intestines from wild type rats, where the staining was located to the apical membrane of enterocytes (Fig. 6C), whereas no specific signal was found in *Slc34a2* homozygous embryos (Fig. 6D) or in samples from wild types in which incubation with the NaPi-IIb antibody was omitted (supplementary Fig. 2).

**Figure 7:**
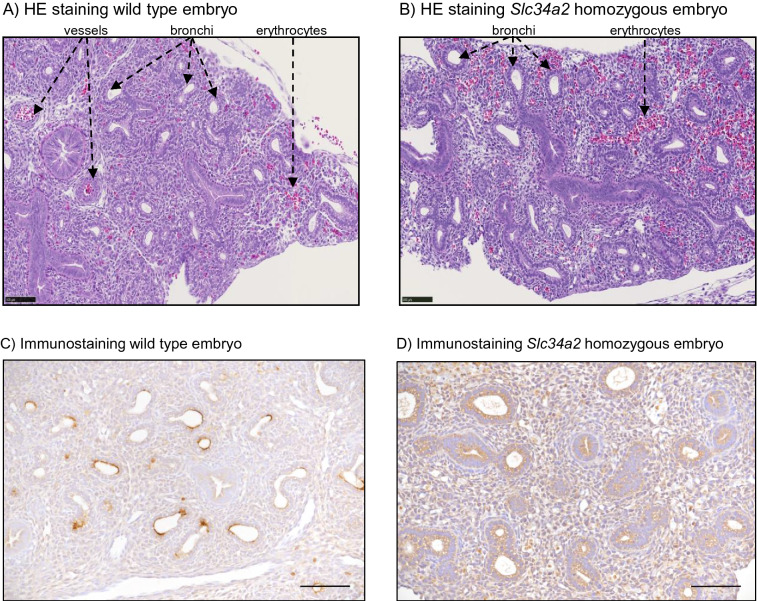
Lungs from (**A**,**C**) wild type and (**B**,**D**) *Slc34a2* homozygous E18 embryos stained with (**A**,**B**) H&E or with (**C**,**D**) a NaPi-IIb antibody. Scale bars: 100 µm in (**A**,**B**); 50 µm in (**C**,**D**).

As expected for their age, the lungs from wild type (Fig. 7A) and homozygous embryos (Fig. 7B) showed typical features of the transition between pseudoglandular to canalicular phases, with extensive airway branching and bronchi formation. NaPi-IIb immunoreactivity was also detected in lungs of wild type embryos, with the protein signal located in the apical membrane of airways as well as in pseudoglandular structures (Fig. 7C), whereas again no specific signal was observed in *Slc34a2* homozygous littermates (Fig. 7D) or in tissue from wild types incubated without primary antibody (supplementary Fig. 2).

**Figure 8:**
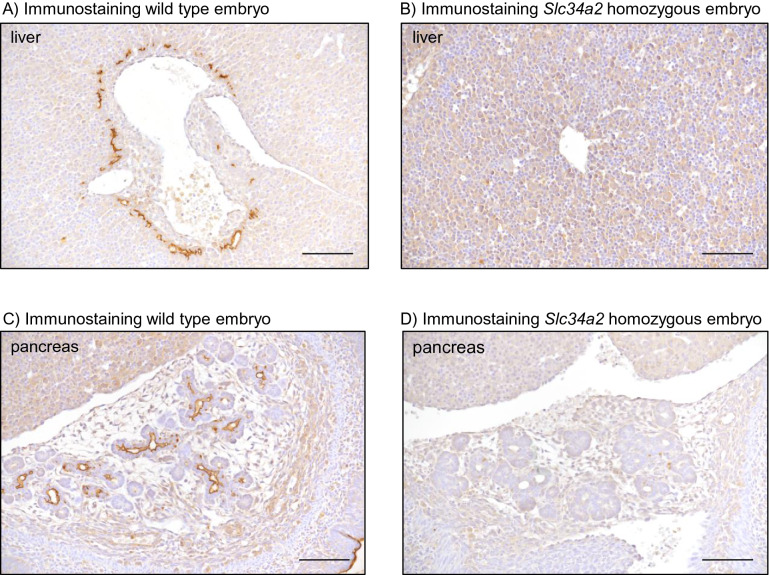
Liver (**A**,**B**) and pancreas (**C**,**D**) from wild type (**A**,**C**) and *Slc34a2* homozygous E18 embryos (**B**,**D**) stained with a NaPi-IIb antibody. Scale bars: 50 µm.

No morphological differences were detected between livers and pancreas from both genotypes. In wild type embryos, expression of NaPi-IIb was observed in canalicular structures around some hepatic blood vessels (Fig. 8A), as well as in the lumen of acini and ductal structures in the pancreas (Fig. 8C). No specific signal was detected in organs from *Slc34a2* homozygous embryos (Fig. 8B,D) or is samples from wild types processed without primary antibody (supplementary Fig. 2).

**Figure 9:**
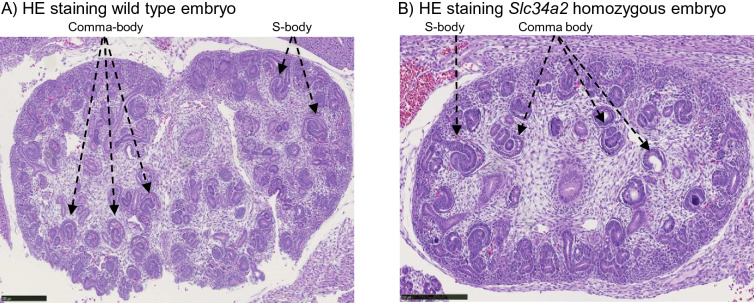
Kidneys from (**A**) wild type and (**B**) *Slc34a2* homozygous E18 embryos stained with H&E. Scale bars: 250 µm.

No differences between both genotypes were observed either regarding their renal development: tubular structures resembling comma-bodies and S-bodies stages of nephron morphogenesis were observed in kidneys from wild type (Fig. 9A) and homozygous *Slc34a2* embryos (Fig. 9B). Occasionally, weak NaPi-IIb protein expression was found in tubular structures of kidneys from wild types but not in *Slc34a2* homozygous samples (data not shown).

## Discussion

Although the kidney has been classically considered as the main organ responsible for the control of plasma Pi levels, more recent data suggest that also the intestine may contribute to this process. Interest in intestinal transport of Pi and its underlying mechanisms is based on several observations, including reports showing that western diets are loaded with far greater than recommend amounts of highly bioavailable Pi^46,47^, that hyperphosphatemia is a risk factor for the development of cardiac disease not only in subjects with impaired renal function as originally thought but also in the normal population^48,49^, and that blocking intestinal absorption could be used as a therapeutic approach to prevent hyperphosphatemia (for review see^50^).

Most of the in vivo information regarding regulation and contribution of particular transporters to Pi balance has been obtained by studies using wild type and genetically modified mouse lines. Thus, whereas a main contribution of NaPi-IIa/*Slc34a1* to renal reabsorption of Pi was already suggested more than 20 years ago when the cotransporter was ablated in mice^51^, human studies corroborating the role of this transporter in humans are very recent^25,26^. However, mice are not always the right experimental model from which to extrapolate findings into Pi handling in humans. For instance, while 2 different mouse lines depleted of NaPi-IIc/*Slc34a3* have no signs for compromised renal Pi handling Pi^52,53^, mutations of this transporter in humans cause a severe derangement of Pi homeostasis, namely HHRH^27–29^. Moreover, constitutive ablation of NaPi-IIb/*Slc34a2* in mice results in embryonic mortality^42^ whereas its absence in humans is not lethal since PAM has been diagnosed in adults and even elderly people^41^. Together, these later findings question the translatability of data obtained from mice to humans.

Here, we describe a rat model constitutively depleted of *Slc34a2*. Although heterozygous rats were indistinguishable from wild type littermates, homozygosity was lethal. Fecal, urinary and plasma levels of Pi were similar in wild type and heterozygous littermates under standard dietary conditions as well as upon 3 days challenging with diets containing low Pi. Furthermore, the basal circulating levels of intact FGF23 and 1,25(OH)_2_ vitamin D_3_, two major Pi-regulating hormones, were also similar in both genotypes and were appropriately regulated in mutant rats in response to reduced dietary Pi. Thus FGF23, a phosphaturic hormone that acts on renal proximal tubules inducing the removal of Na^+^/Pi cotransporters^54–56^, was properly and similarly downregulated in wild type and heterozygous rats fed on low Pi. In contrast 1,25(OH)_2_ vitamin D_3_, a steroid hormone that targets the intestine promoting the expression of NaPi-IIb therefore stimulating active intestinal absorption of Pi^30,57^, was similarly upregulated in both genotypes. Together, these results suggest that systemic Pi balance in rats is not compromised by depletion of a single *Slc34a2* allele.

Analysis of embryos at stage E18 indicated that although all three genotypes were detected at about the expected Mendelian ratio, *Slc34a2* homozygous embryos were clearly smaller than wild type and heterozygous embryos, with no differences between the two later ones. A histological analysis in H&E stained sections revealed that all organs were smaller in homozygous embryos than in wild type littermates, but without evident anatomical signs of organ malformation or tissue damage (i.e. necrosis or haemorrhages). The intestine of wild type and homozygous embryos had an appearance consistent with the onset on villus morphogenesis, in agreement with the expected intestinal formation in rodents. This process is initiated by clustering of mesenchymal cells below the undifferentiated pseudostratified epithelia, with clustering driving the projection of the epithelial cells into the lumen^58^. In mice whose pregnancy is shorter than rats, intestinal villi morphogenesis proceeds between E14.5-E16.5, though formation of mature crypts at the intervilli domain is not completed until P14^58^, a time frame consistent with the morphological features found in E18 rat embryos. At this developmental stage, clear expression of NaPi-IIb was observed at the apical membrane of intestinal epithelial cells in wild type embryos. In humans, the lung is the main organ affected by mutations of *Slc34a2/*NaPi-IIb, and the PAM phenotype is mimicked in mice with inducible full-body *Slc34a2* ablation^16,17,43^. Lungs from E18 wild type and *Slc34a2* homozygous embryos have features consistent with the normal transition from pseudoglandular (E9.5–E16.5 in mice) to canalicular phases (E16.5–E17.5 in mice), with extensive airway branching and bronchi formation^59^. In wild type E18 embryos, expression of NaPi-IIb was detected in the apical membrane of epithelial cells lining airways as well as in some pseudoglandular structures. In the murine adult lung, NaPi-IIb expression was reported in type II alveolar epithelial cells^20^ which, in addition to their proposed role in clearing Pi from the intra-alveolar space, are responsible for the production, secretion and at least partial recycling of surfactants. However, alveolarization takes place only in neonates (P1–P14 in mice)^59^. As for intestine and lung, the morphology of kidneys was similar in wild type and *Slc34a2* homozygous embryos. In mice, metanephric kidney development starts at E10.5 but is not completed until P14^60^. Renal development begins with the formation of the uretric bud and its branching, followed by mesenchymal-to-epithelial transition of cells at the tips of branching. Here, kidneys from both genotypes contained numerous comma- and S-shape bodies that represent progressive nephron developmental stages. Expression of NaPi-IIb was detected in the lumen of few tubular structures. In adult mice, NaPi-IIb is localized in the loop of Henle^22^, thus the structures stained here in embryonic rat kidney could eventually develop into loops of Henle. The concentration of Pi in the bile is about two orders of magnitude lower than in plasma; NaPi-IIb, the expression of which was reported at the canalicular membrane of hepatocytes and BBM of cholangiocytes in adult rats, has been proposed to mediate reabsorption of Pi from the primary bile^21^. Here, NaPi-IIb protein expression was detected in canalicular structures around blood vessels in the liver of rat embryos. In addition, lumen of acini and ductal structures in the pancreas were also positive for NaPi-IIb; to our knowledge the function of the transporter in pancreatic tissue remains unknown.

In summary, homozygous ablation of NaPi-IIb/*Slc34a2* leads to embryonic lethality not only in mice but also in rats. However, whereas NaPi-IIb deficiency is associated with early embryonic lethality in mice, rat homozygous embryos developed to late stages of embryogenesis. While identifying the cause of the lethality of homozygous *Slc34a2* depleted rats is beyond the scope of this work, our data indicate that the cotransporter plays an essential role during embryonic and perinatal development in both rodent models. This is in contrast to humans, were homozygous mutations of *SLC34A2* predicted to result in complete loss of function have been identified in elderly PAM patients. Though our findings do not allow conclusions as to whether rats are a better model than mice to study intestinal Pi absorption, they indicate that differences in Pi and Pi-transporter physiology across species should be considered for translational research.

## Material and methods

### Generation of Slc34a2^+/−^ rats

Rats with constitutive depletion of NaPi-IIb/*Slc34a2* were generated by Charles River using a Crispr-Cas approach. Endonuclease activity produced a 262 bp truncation resulting in removal of the whole exon 2 (115 bp) plus the first bps from intron 2–3. Exon 2 contains the initiator ATG and encodes for the first 37 amino acids, therefore its removal is expected to abolish expression of the transporter. Genotyping was performed by PCR amplification of genomic DNA isolated from ear plugs, in the presence of a forward primer annealing within intron 1–2 (TGCAGCCAGTGAAGACCATT) and reverse primer annealing within intron 2–3 (AGGAGTCCCGCTGTCATTTG). Reactions were expected to produce amplicons of 357 bp in wild types (WT) and 95 bp in mutant rats.

### Animal handling and collection of samples

Experiments were performed in 3–4 months old wild type and heterozygous *Slc34a2* male rats. Animals fed standard diet (KLIBA, SA: 0.8% Pi, 1% Ca^++^ and 800 u/Kg vitamin D_3_) were housed individually for 24 h in metabolic cages (Tecniplast) for the collection of feces and urine. Rats were then transfer to normal cages and fed for 3 additional days on low phosphate diet (KLIBA, SA: 0.1% Pi, 1% Ca^++^ and 800 u/Kg vitamin D_3_). During the last 24 h, they were transferred back to metabolic cages. At termination, animals were anaesthetized with isoflurane and heparinized-blood as well as intestinal mucosa from duodenum and jejunum were collected. Tissue samples as well as aliquots of urine and plasma were immediately frozen in liquid nitrogen and stored at − 80 °C until use. For embryonic analysis, 3 heterozygous breedings were set up and females were sacrificed at day 18 post-coitus. Embryos were dissected and biopsies for genotyping were collected from toes upon which embryos were fixed in 4% paraformaldehyde. The study was carried out in compliance with the Animal Research: Reporting of In Vivo Experiments (ARRIVE) guidelines. Breeding and all experimental protocols complied with the Swiss Animal Welfare laws and had been previously approved by the ethics committee of Zurich Veterinary Office (Kantonales Veterinäramt Zürich; license number 156/2016).

### Quantification of Pi and Ca^2+^ in urine, stool, plasma and amniotic fluid

Prior determinations, stool samples were processed as indicated previously^44^. The concentration of Pi in all there samples was quantified with the Fiske Subarow method (Sigma Diagnostics), whereas Ca^2+^ was measured with the QuantiChrom Calcium assay kit (Bio-Assay Systems). The urinary excretion of both electrolytes was normalized to the creatinine output, that was determined according to the Jaffe method (Wako Chemicals).

### Quantification of FGF23 and 1,25(OH)_2_ vitamin D_3_ in plasma

Plasma levels of intact FGF23 were measured by ELISA (Immutopics) following the manufacturer’s protocols. The concentration of 1,25(OH)_2_ vitamin D_3_ was quantified by radioimmunoassay (Immunodiagnostic System).

### Western blots

Samples of total membrane (20 µg) isolated from mucosa of duodenum or jejunum were separated into SDS/PAGE gels and transferred to PVDF membranes (Millipore) by standard procedures. Non-specific antibody binding was prevented by incubating the membranes for 30 min at room temperature in 5% non-fat milk powder in TBS. Then, membranes were incubated overnight at 4 °C with a primary polyclonal antibody against rat NaPi-IIb^32^ followed by 2 h incubation at room temperature with HRP-conjugated anti rat secondary antibody (GE Healthcare; dilution 1: 5000). Upon short exposure to HRP substrate (Millipore), antibody-related signals were collected with the LAS-4000 luminescent image analyzer (Fujifilm) and further quantified (ImageJ). Upon stripping for 30 min at room temperature in a solution containing 25 mM glycine, 1% SDS, pH 2, membranes were incubated with an β-actin antibody (Sigma; dilution 1: 20,000) and processed again as indicated above.

### Histological and immunohistochemical evaluations

After fixation, whole embryos were cut longitudinally and the two midsagittal halves were placed in a cassette and embedded in paraffin wax. Sections with 3–5 µm thickness were stained with hematoxylin and eosin (H&E) or subjected to immunohistochemical evaluation. Immunohistochemical analysis for NaPi-IIb was performed in the Dako autostainer system (Dako, Glostrup, Denmark) using the primary polyclonal antibody against rat NaPi-IIb indicated above. Briefly, upon antigen retrieval in citrate buffer (pH 6) at 98 °C for 20 min, sections were incubated overnight at 4 °C in the presence of the primary antibody (dilution 1:500). Upon application of EnVision-HRP anti-rabbit secondary antibody (Dako K4003) DAB substrate buffer (K3468) was used for the detection. As negative control, samples from wild type embryos were processed in parallel omitting the incubation with the primary antibody.

### Statistical analysis

Differences between groups were analyzed by t-test (2 groups) or Anova/ Bonferroni’s test (multiple groups), as indicated. *P* < 0.05 was considered significant**.** All data are shown as mean ± SEM.

## Supplementary Information

Supplementary Information
