# Supplementary material for: Constitutive depletion of Slc34a2/NaPi-IIb in rats causes perinatal mortality
Source: Sci Rep. 2021 Apr 12;11:7943. doi: 10.1038/s41598-021-86874-z (PMC8042035; doi:10.1038/s41598-021-86874-z)
Supplement: Supplementary file 1 — Supplementary Information [file 41598_2021_86874_MOESM1_ESM.pdf]

Constitutive depletion of *Slc34a2*/NaPi-IIb in rats causes perinatal mortality

Eva Maria Pastor-Arroyo, Josep M Monné Rodriguez, Giovanni Pellegrini, Carla Bettoni, Moshe Levi, Nati Hernando and Carsten A. Wagner

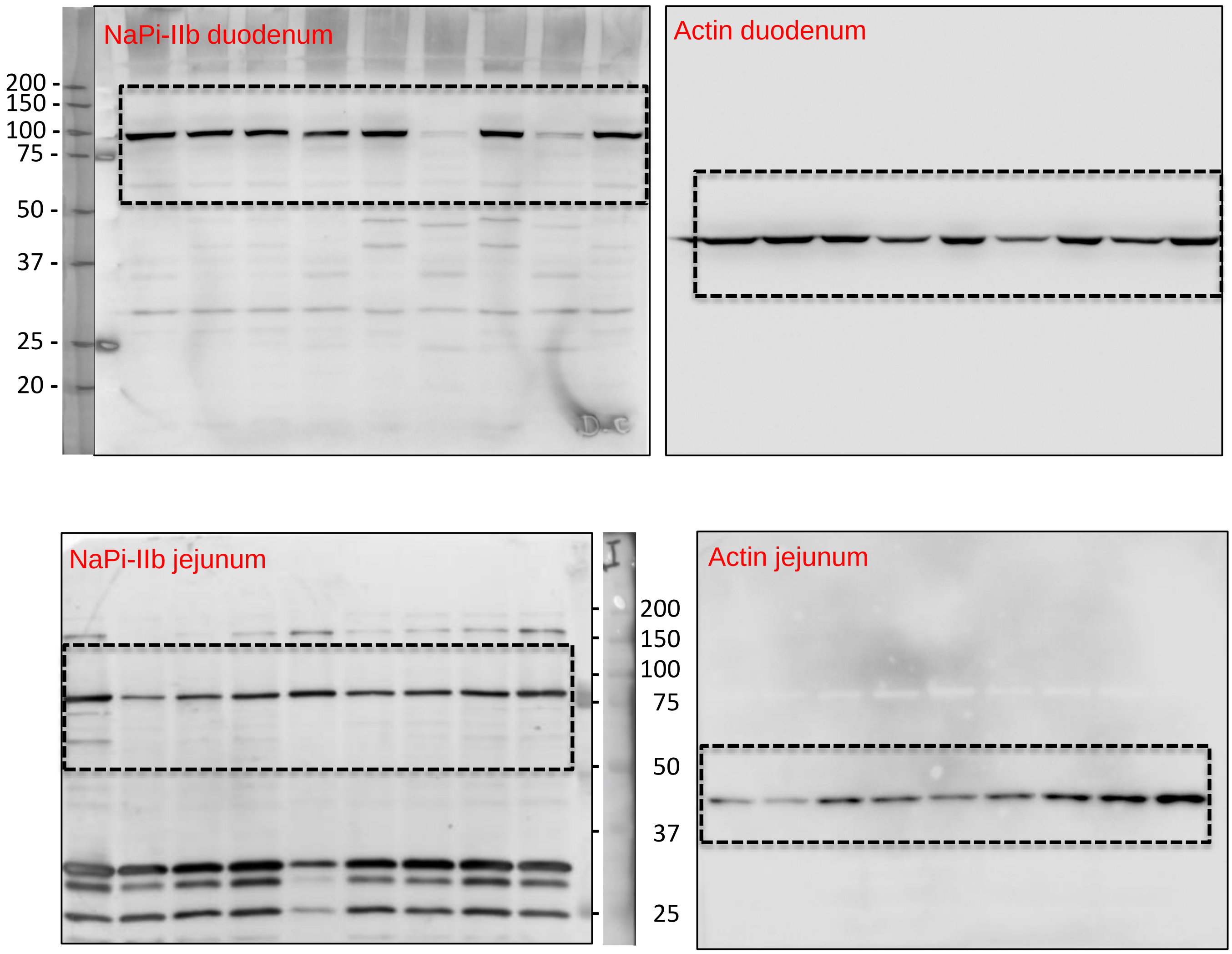

Supplementary Figure 1. Original Western blot images obtained in samples from duodenum and jejunum incubated with antibodies against NaPi-IIb and actin. Rectangles indicate the areas shown in Figure 4.

Constitutive depletion of *Slc34a2*/NaPi-IIb in rats causes perinatal mortality

Eva Maria Pastor-Arroyo, Josep M Monné Rodriguez, Giovanni Pellegrini, Carla Bettoni, Moshe Levi, Nati Hernando and Carsten A. Wagner

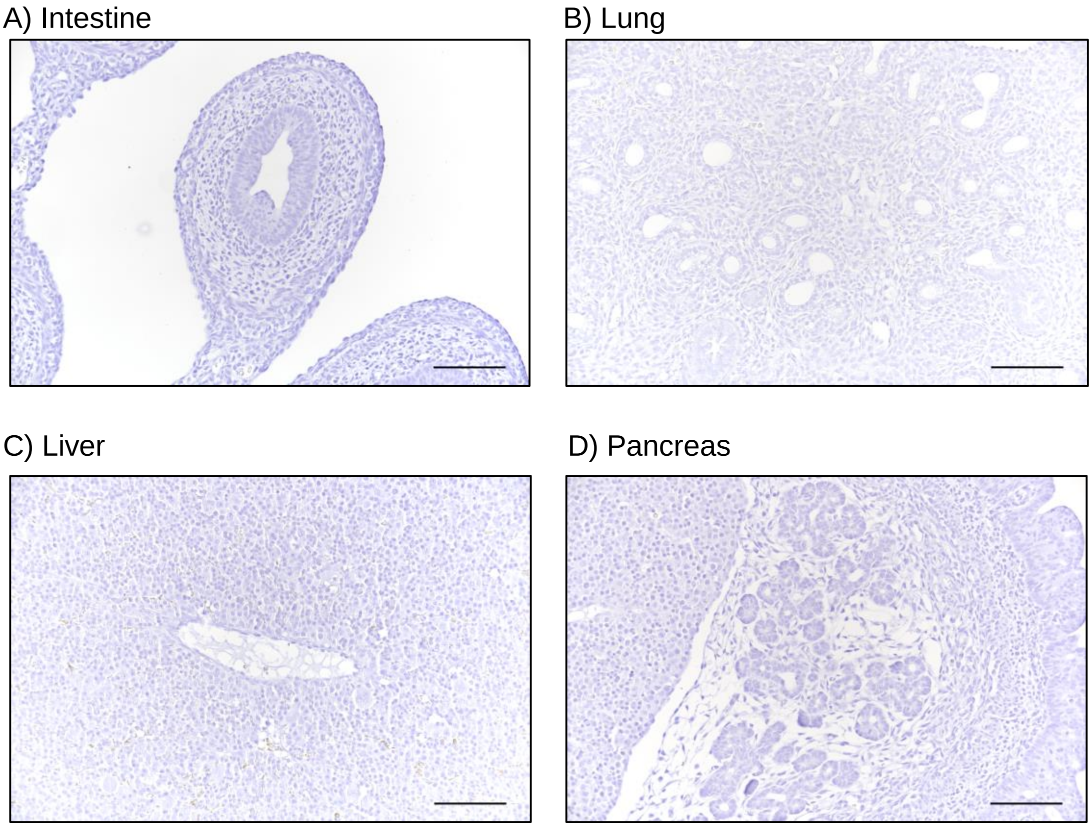

Supplementary Figure 2. Intestine (A), lung (B), liver (C) and pancreas (D) of wild type E18 embryos processed for immunohistochemistry in the absence of primary (NaPi-IIb) antibody. Scale bars: 50 µm.
